# The Comparison of the Podocyte Expression of Synaptopodin, CR1 and Neprilysin in Human Glomerulonephritis: Could the Expression of CR1 be Clinically Relevant?

**Published:** 2009-03

**Authors:** Anna Kubiak-Wlekły, Agnieszka Perkowska-Ptasińska, Paweł Olejniczak, Aleksandra Rochowiak, Elżbieta Kaczmarek, Magdalena Durlik, Stanisław Czekalski, Zofia I. Niemir

**Affiliations:** 1 *Department of Nephrology, Transplantology and Internal Diseases, Karol Marcinkowski University of Medical Sciences, Poznan, Poland;*; 2 *Department of Transplantation Medicine and Nephrology, Transplantation Institute, Warsaw, Poland;*; 3 *Department of Clinical Pathomorphology, Karol Marcinkowski University of Medical Sciences, Poznan, Poland*

**Keywords:** glomerulonephritis, podocytes, CR1, neprilysin, synaptopodin

## Abstract

Podocytes are considered as the most important cells that determine loss of structure and function of the glomerular filter. We compared the expression of three podocyte markers, i.e.: synaptopodin (SYN), CR1 and neprilysin (NEP) in 107 patients with different forms of glomerulonephritis (GN) and 5 normal kidneys (NK). A quantitative immunohistochemistry was applied to evaluate the expression of podocyte proteins. The results were related with serum creatinine (Scr), estimated glomerular filtration rate (eGFR) and urinary protein.

We observed the reduction in the podocyte expression of NEP, SYN and CR1 in proliferative and non-proliferative forms of GN. Interestingly, in mesangial proliferative GN (MesPGN), the expression of SYN and CR1 was lower in IgA-MesPGN than in non-IgA-MesPGN (*p*<0.005 and *p*<0.02, respectively). In all the patients, the expression of NEP and SYN was positively related (r=0.53, *p*=0.02) as that of NEP and CR1 (r=0.39, *p*=0.04). Yet, clinical correlations with Scr (r=-0.33, *p*=0.03) and eGFR (r=0.26, *p*=0.05) were obtained only with respect to CR1.

In conclusion, SYN, CR1 and NEP may be used as markers of podocyte loss in patients with GN. However, in agreement with previous studies, the clinical relevance draws a special attention to the expression of CR1.

## INTRODUCTION

Podocytes turned out to be the most important cells that determine the loss of structure and function of the glomerular filter (1). There are many podocyte markers. Among them are synaptopodin (SYN), podocalyxin, Wilm’s tumour protein-1, neprilysin (NEP, CD10 or CALLA-common acute lymphoblastic leukemia antigen), glomerular epithelial protein-1, and C3b receptor for complement (C3bR or CR1) (2-5).

SYN is an actin-associated protein localized in the foot processes of podocytes and dendritic spines of telencephalic synapses. During kidney development, SYN first appears at the capillary loop stage and plays an important role in the regulation of foot processes shape and mobility. The expression of SYN indicates the advanced cytoskeleton development and therefore it is considered an important marker of the phenotypic maturity of podocytes and neurons (4).

CR1 is localized, apart from podocytes, on monocytes, erythrocytes, neutrophils, eosinophils, and B-lymphocytes. It is an integral membrane glycoprotein of 210 kDa, which during kidney development first appears at the capillary loop stage (6). CR1 is a co-factor for factor I, a plasma serine proteinase that cleaves C3b and C4b (5). Ultrastructural examination showed that CR1 is homogeneously distributed on the plasma membrane of the main body, trabecules, and pedicels of the podocyte (7).

NEP is expressed in normal granulocytes and malignant hematopoietic cells and in epithelial cells of many organs including liver, kidneys, stomach, lungs, urinary bladder, and uterus (8). In kidneys, it is expressed in podocytes, renal proximal tubular epithelium and in smooth muscles of the vessels. During the kidney development, its expression first appears in the S-shaped body stage (6, 9). NEP is the first podocytic antigen, which has been shown to induce human membranous GN (9).

Decreased expression of SYN, CR1 and NEP on podocytes has been found in a variety of glomerular diseases (2, 7, 10-16). There are, however, only few studies aimed at the analysis of more than one podocyte marker in different forms of human glomerular disease (2, 10). Of interest, decreases in the expression of CR1 and NEP were associated with renal dysfunction, histopathological damage and poor renal prognosis in IgA nephropathy (10, 13). Other studies demonstrated that reduced expression of SYN in minimal change disease (MCD) and focal-segmental glomerulosclerosis (FSGS) was related with a poor response to steroid therapy (17, 18).

Thus, it seemed reasonable to perform a parallel analysis of the podocyte expression of NEP, CR1 and SYN in various forms of primary and secondary GN. We also addressed the question of a potential relationship between serum creatinine (Scr), estimated glomerular filtration rate (eGFR) and/or proteinuria and the expression of each marker.

## MATERIAL AND METHODS

One hundred seven patients with biopsy-proven GN were included in the study: 92 with primary GN and 15 with secondary GN. All examined subjects have given their informed consent. The Poznań University of Medical Sciences committee on human research has approved the study protocol. The diagnosis of GN was based on conventional morphological and immunopathological examinations of renal biopsy specimens. Out of 70 patients with primary proliferative GN (PGN), 65 had mesangial proliferative GN (MesPGN) and five membranoproliferative GN (MPGN). Patients with MesPGN presented morphologically with varied degrees of mesangial expansion/proliferation and/or sclerosis along with variable severity of tubulointerstitial lesions. Based on the results of the immunopathological examination they were categorized as IgA mesangial proliferative GN (IgA-GN; 45 patients) or non-IgA-MesPGN (20 subjects with dominant mesangial deposits of IgM, C_3_, C_4_, and C1q). In the group of 22 patients with primary non-proliferative forms of GN (NPGN), 11 had idiopathic membranous GN (IMGN), 9 focal and segmental glomerulosclerosis (FSGS), and two minimal change diseases (MCD). The group with secondary forms of GN consisted of 10 patients with lupus nephritis (LGN; two with class III, 4 with class IV, and 4 with class V) and 5 with extracapillary GN (ExGN; 2 - cANCA-positive and 3 - ANCA-negative). Morphological, clinical and laboratory data of the patients are summarized in Table 1. All the patients presented with variable degrees of proteinuria and erythrocyturia, and normal or impaired renal function. In the patients with secondary forms of GN, a detailed clinical history, examination and laboratory tests confirmed the systemic disease.

**Table 1:** Clinical and morphological data of patients at the time of renal biopsy

Morphology	No. of Patients	Sex (M/F)	Age yr Mean ± SD	Duration of Symptoms Months Mean ± SD	Serum Creatinine (μmol/L Mean ± SD)	eGFR mL/min/1.73m^2^ Mean ± SD	Urinary Protein g/24h Mean ± SD

**PRIMARY GN**							
IgA-MesPGN	45	20/25	29.55 ± 10.14	14.33 ± 12.28	93.70 ± 140.55	64.91 ± 50.64	2.29 ± 1.64
Non- IgA-MesPGN	20	12/8	32.47 ± 14.93	9.25 ± 5.32	109.61 ± 27.40	53.11 ± 25.55	3.38 ± 3.43
MPGN	5	4/1	52.00 ± 9.90	5.48 ± 3.06	221.00 ± 62.76	21.50 ± 12.87	3.75 ± 0.35
MCD	2	0/2	44.00 ± 32.52	3.00 ± 1.40	88.40 ± 12.37	81.12 ± 21.40	4.10 ± 0.56
FSGS	9	3/6	35.00 ± 20.57	8.58 ± 5.25	205.08 ± 66.30	25.39 ± 8.35	3.14 ± 3.20
IMGN	11	6/5	38.00 ± 13.81	6.35 ± 2.15	88.40 ± 49.50	65.95 ± 25.48	6.18 ± 8.04
**SECONDARY GN**							
**LGN**							
Class III	2	0/2	30.00 ± 1.41	6.5 ± 2.12	137.02 ± 61.88	41.73 ± 11.52	2.00 ± 0.70
Class IV	4	0/4	41.33 ± 17.03	17.35 ± 13.25	114.92 ± 22.98	47.90 ± 16.23	2.80 ± 2.97
Class V	4	0/4	28.33 ± 2.08	7.52 ± 3.15	73.37 ± 9.72	86.81 ± 12.53	3.06 ± 3.18
**ExGN**							
ANCA (+)	2	2/0	47.00 ± 1.41	0.75 ± 0.35	517.14 ± 330.61	5.33 ± 2.13	3.00 ± 2.12
ANCA (-)	3	3/0	59.50 ± 10.60	1.67 ± 0.58	689.52 ± 74.25	5.62 ± 0.87	1.80 ± 0.42

IgA-MesPGN, mesangial proliferative IgA glomerulonephritis; Non-IgA-MesPGN, mesangial proliferative non-IgA glomerulonephritis; MPGN, membrano-proliferative glomerulonephritis; IMGN, idiopathic membranous glomerulonephritis; FSGS, focal-segmental glomerulosclerosis, MCD, minimal change disease; LGN, lupus glomerulonephritis; ExGN, extracapillary glomerulonephritis; ANCA (+), ANCA-positive GN; ANCA (-), ANCA-negative GN; eGFR, estimated glomerular filtration rate according to the Modification of Diet in Renal Disease formula.

The expression of NEP, CR1 and SYN was examined by immunohistochemistry on acetone-fixed biopsy specimens using the APAAP method as previously described (11). Biopsy specimens containing at least five glomeruli were qualified for the study. Mouse anti-human monoclonal antibodies against NEP (Dako, Glostrup, Denmark), CR1 (Dako, Glostrup, Denmark) and SYN (Progen, Heidelberg, Germany) were applied in this study. Five normal appearing fragments of kidney tissue surrounding the removed tumour served as controls. Control experiments were conducted by omitting the incubation with the primary antibody, as well as with substitution of the primary antibody with non-immune murine serum.

**Figure 1:**
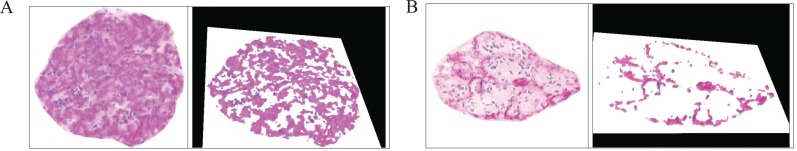
A, An extracted NEP stained normal glomerulus (left) and a spatial visualization of segmented positive NEP reaction (podocytes’ marker); B, An extracted NEP stained glomerulus in MPGN (left) and a spatial visualization of segmented positive NEP reaction (magnification 400×).

Morphometric measurements of the expression of NEP, CR1 and SYN were performed using an originally designed computer technique for quantitative immunohistochemistry programmed in C++ language (19). The method is presented in the Fig. 1A and 1B. Briefly, colour light micrographs were extended to three-dimensional images by introducing the intensity of colour reaction as the third dimension. Then, the colour immunohistochemical reaction was exposed on the three-dimensional view by reducing the scenery behind the background. Next, filters of colour, brightness and saturation were fixed for image series acquired from each specimen, and colours representing the immunohistochemical reaction were extracted. The expression of extracted reaction was derived from orthogonal projection of its spatial representation onto the plane by determining the reaction area. The relative area of reaction per glomerulus was calculated as the ratio of the reaction area and glomerular area (in percents). Finally, the mean expression score (MES) was calculated for the each patient by summarizing the values obtained for all glomeruli and dividing this amount by the number of glomeruli.

The obtained results were then statistically analyzed by using Statistica v. 7.1 (Statsoft Inc.). First, a consistency of the results with Gaussian distribution was verified by using Shapiro-Wilk normality test. Then, one-way analysis of variance (ANOVA) was performed with a minimal significant difference post-hoc test. Results were accepted as significantly different at significance level *p*<0.05. Spearman’s correlation coefficients between the expression of NEP, CR1 and SYN, as well as between the expression of these peptides and Scr, eGFR and urinary protein of examined patients were calculated. We used the Modification of Diet in Renal Disease (MDRD=186.3 × Scr [mg/dL]^-1.154^ × age [year]^-0.203^ × 1.212 if African-American × 0.742 if female) equation to calculate eGFR (20).

## RESULTS

**Figure 2:**
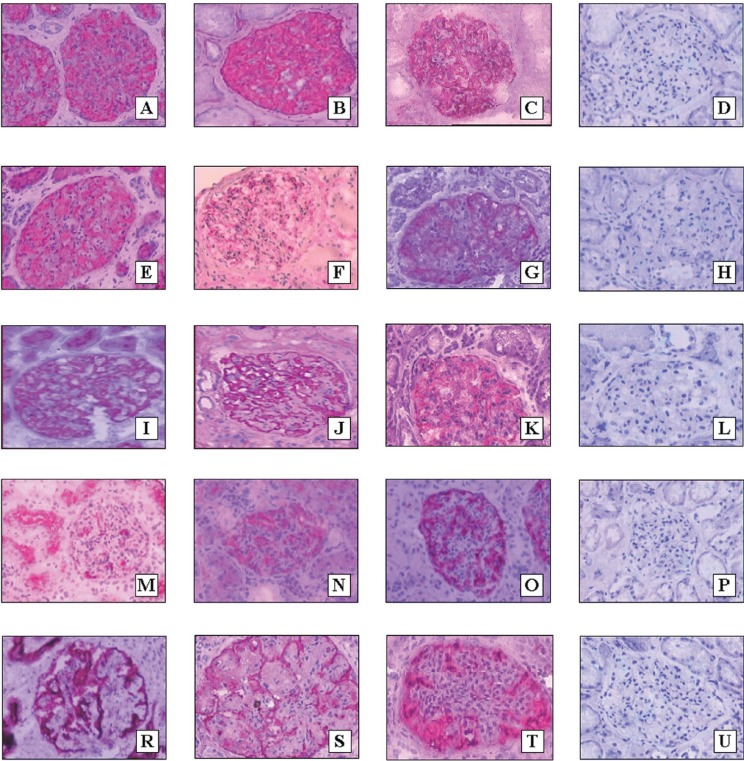
The expression of NEP (A, E, I, M, R), synaptopodin (B, F, J, N, S) and CR1 (C, G, K, O, T) in NK (A, B, C), IMGN (E, F, G), MesPGN with moderate proliferation of mesangial cells (I, J, K), MesPGN with high proliferation of mesangial cells (M, N, O) and MPGN (R, S, T). The pictures D, H, L, P, and U represent negative controls (D to A, H to E, L to I, P to N, and U to T) (magnification 400×).

In NK, the expression of NEP, CR1 and SYN was confined to the outer aspects of glomerular capillaries (podocytes). Their MES were 17.46% ± 4.54%, 15.71% ± 7.25% and 18.00% ± 4.75%, respectively. Examples of the glomerular expression of NEP, CR1 and SYN in NK and different morphological forms of GN are presented in Fig. 2.

**Figure 3:**
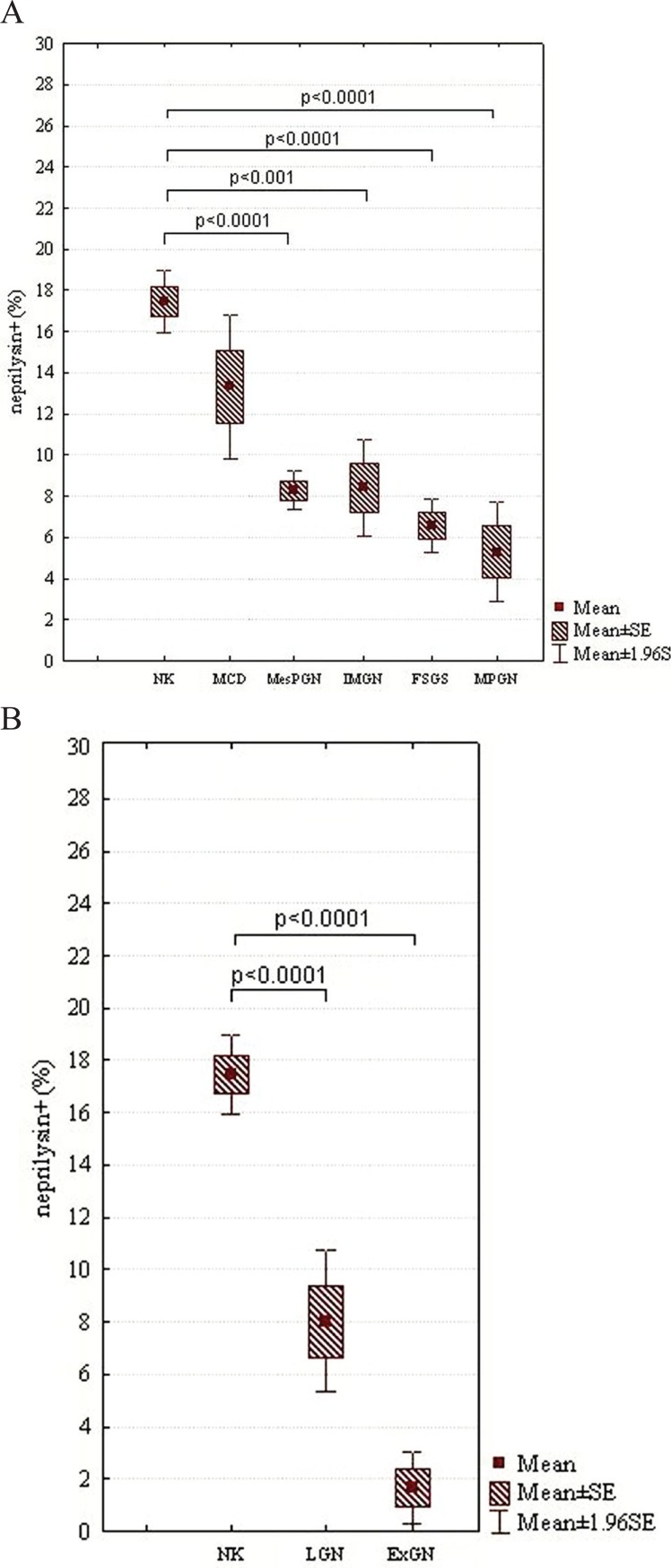
A, MES of NEP in NK and different morphological forms of primary GN, which are arranged according to an expected renal prognosis (from the best to the most serious one); B, MES of NEP in NK and secondary GN, i.e., LGN and ExGN; SE, standard error of mean.

**Figure 4:**
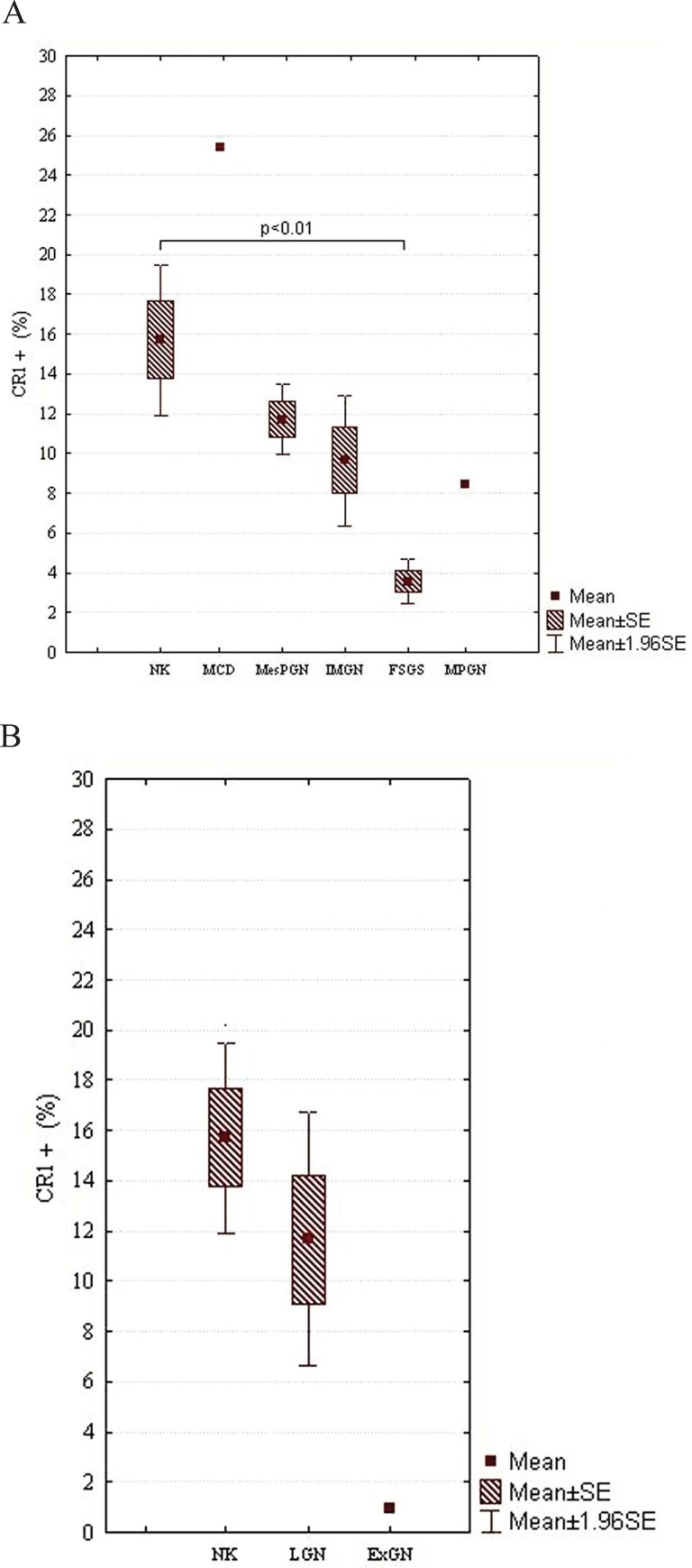
A, MES of CR1 in NK and different morphological forms of primary GN, which are arranged according to an expected renal prognosis (from the best to the most serious one); B, MES of CR1 in NK and secondary GN, i.e., LGN and ExGN; SE, standard error of mean.

**Figure 5:**
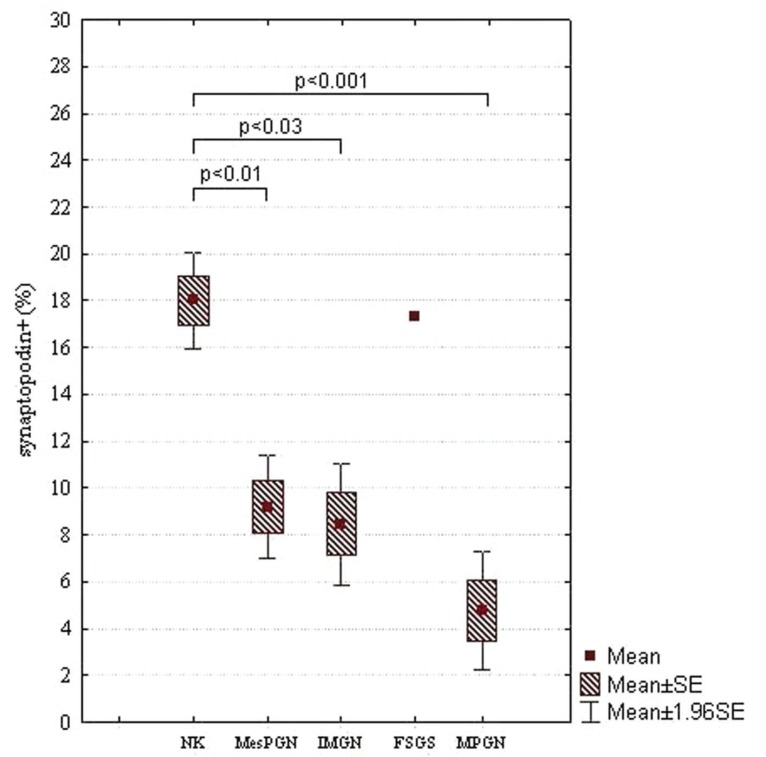
MES of synaptopodin in NK and different morphological forms of primary GN, which are arranged according to an expected renal prognosis (from the best to the most serious one); SE, standard error of mean.

In the case of NEP, there were statistically significant differences between NK and other subjects examined: FSGS (*p*<0.0001), IMGN (*p*<0.001), MPGN (*p*<0.0001), MesPGN (*p*<0.0001), ExGN (*p*<0.0001), and LGN (*p*<0.0001) (class III - 8.98% ± 0.68%, class IV - 9.80% ± 5.90%, class V - 14.61% ± 3.22%). The statistical summary of the expression of NEP in NK and different morphological forms of GN is presented in Figs 3A and 3B. The obtained results were arranged according to an expected renal prognosis. As regards CR1, similarly to NEP, the lowest values of MES were noted in ExGN and FSGS, where the highest proliferation or advanced sclerotic lesions could be observed. In the other groups of patients, the values of MES were higher (IMGN 9.64% ± 5.77%; MesPGN 11.71% ± 5.88% and LGN 11.68% ± 5.74%), but statistical significance reached only the difference between NK and FSGS (*p*<0.01). These data are presented in Figs 4A and 4B. The analysis of SYN expression revealed statistically significant differences between NK and IMGN (*p*<0.03), MPGN (*p*<0.001) and MesPGN (*p*<0.01). The above data are presented graphically in the Fig. 5.

**Figure 6:**
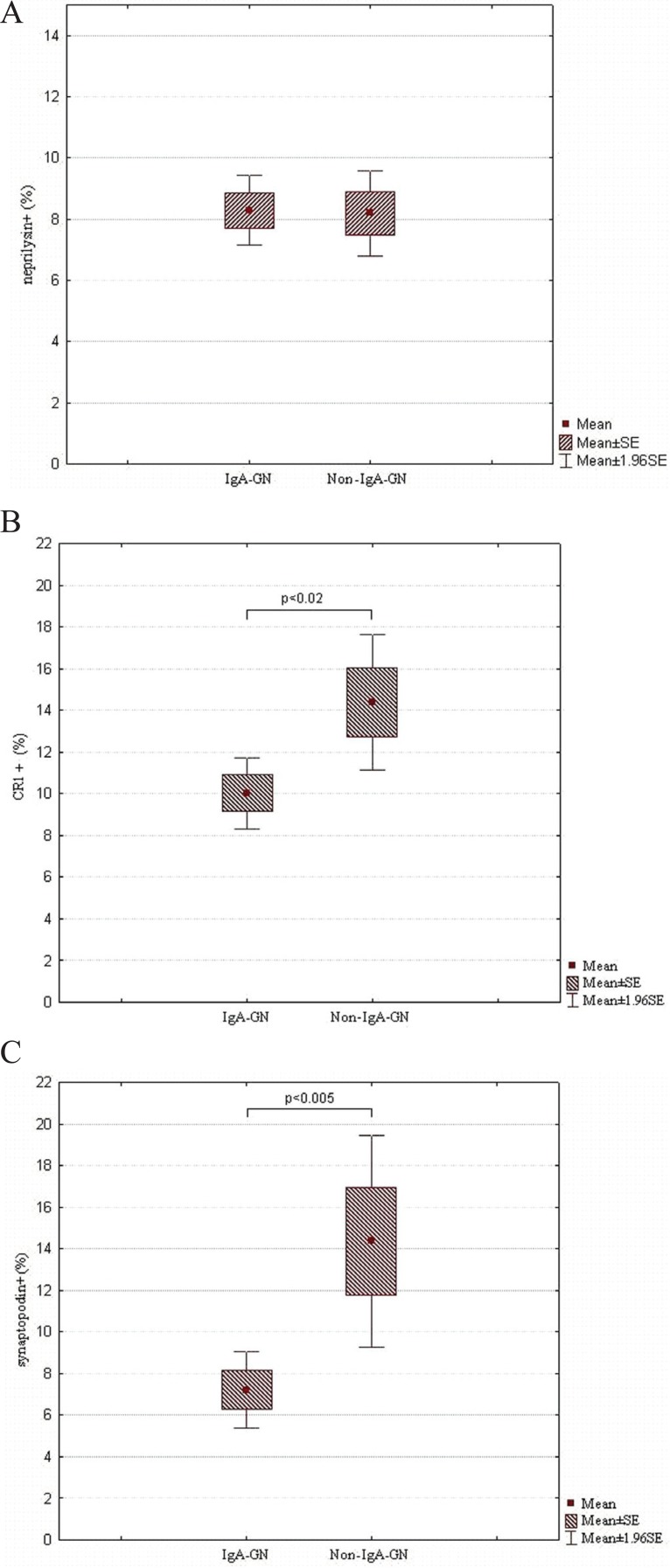
A, MES of NEP in IgA-GN and non-IgA-GN MesPGN; B, MES of CR1 in IgA-GN and non-IgA-GN MesPGN; C, MES of synaptopodin in IgA-GN and non-IgA-GN MesPGN; SE, standard error of mean.

Patients with IgA-GN and those presenting non-IgA MesPGN did not differ with respect to the NEP expression (Fig. 6A). Interestingly, however, patients with IgA-GN showed statistically lower expression of CR1 than those with non-IgA-GN (10.03% ± 4.53% vs. 14.38 ± 6.87; *p*<0.02) (Fig. 6B). The same concerned the expression of SYN (IgA-GN - 7.22% ± 4.28%; non-IgA-GN - 14.36% ± 7.33%; *p*<0.005) (Fig. 6C).

**Figure 7:**
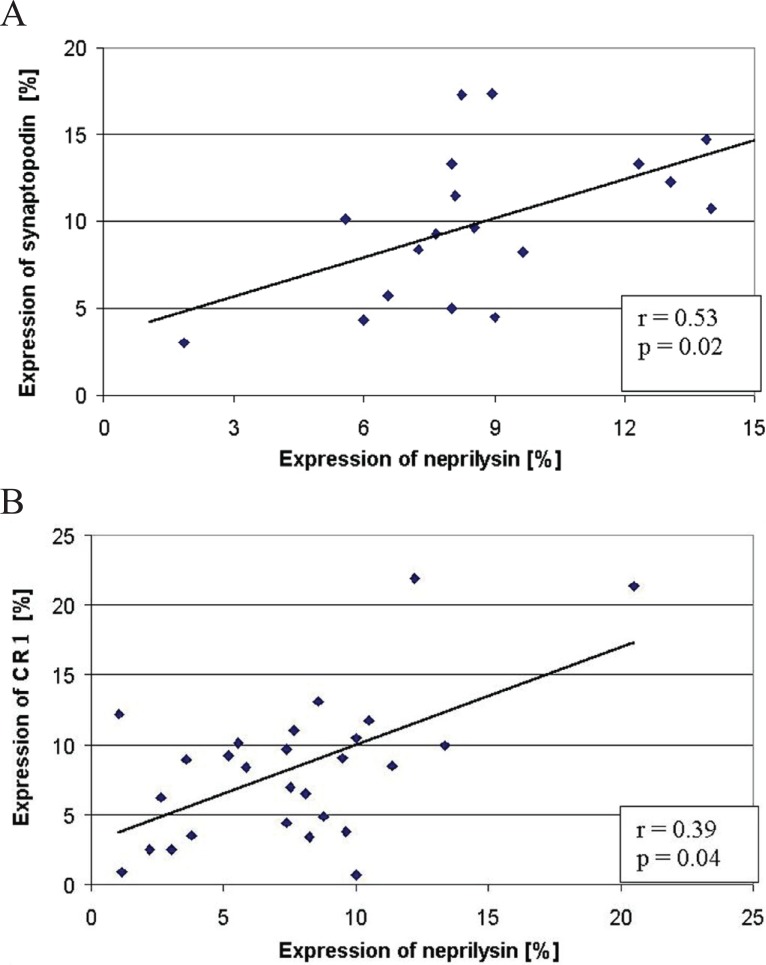
The relationship between the expression of NEP and synaptopodin (A) and between the expression of NEP and CR1 (B) in the examined patients.

When looking for a relationship between the podocyte markers in the whole population of patients examined, we found a significant correlation between the expression of NEP and SYN (r=0.53, *p*=0.02) (Fig. 7A), as well as between NEP and CR1 (r=0.39, *p*=0.04) (Fig. 7B). No relationship could be stated between the expression of CR1 and SYN.

**Figure 8:**
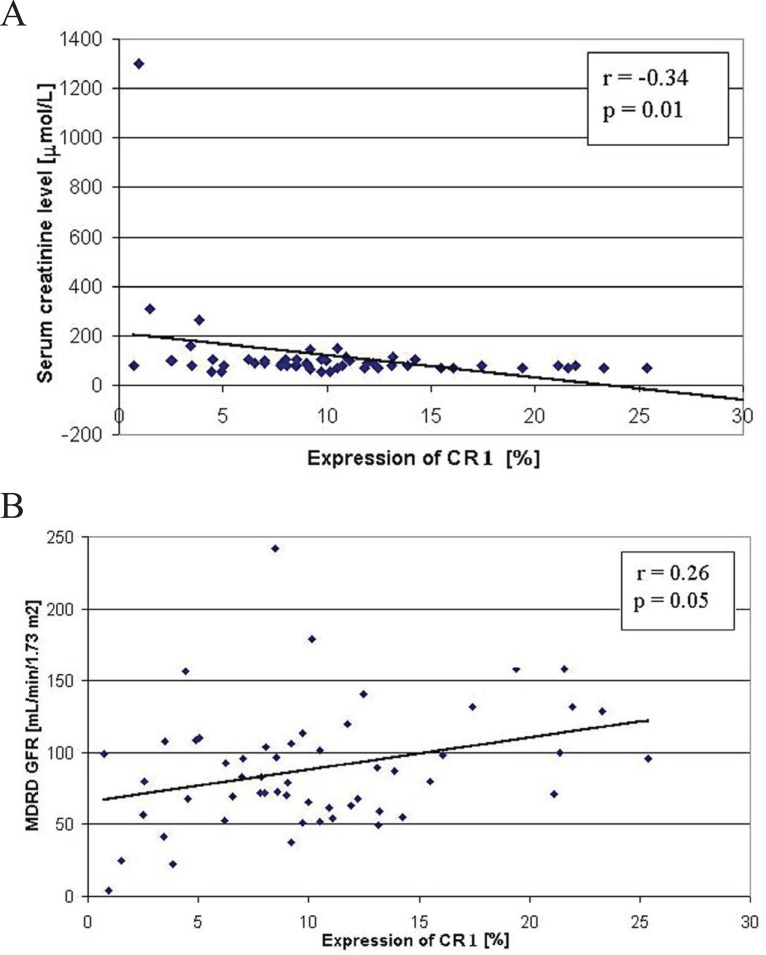
(A) The relationship between the expression of CR1 and serum creatinine level (A) and between the expression of CR1 and eGFR (according to the MDRD equation) (B) of the examined patients.

We found an inverse relationship between the podocyte CR1 expression and Scr of the examined patients (r=-0.34, *p*=0.01) (Fig. 8A). Also, a low positive correlation between CR1 expression and eGFR (r=0.26, *p*=0.05) (Fig. 8B) could be noted. However, no relationship between the expression of CR1 and urinary protein, as well as between the expression of NEP and SYN and urinary protein, Scr or eGFR of the examined patients was obtained.

## DISCUSSION

In this study, the expression of SYN was decreased in all subgroups of patients examined. The reduction in SYN expression was mainly noted in cases with marked glomerular cell proliferation and/or advanced sclerotic alterations. These results confirm our previous observations (11, 12), but they are in disagreement with the data published by the other authors. Barisoni *et al*. (2) noticed the reduction in the SYN expression in glomerulopathies with phenotypic dysregulation of podocytes, i.e. in idiopathic collapsing FSGS and HIV-associated nephropathy, whereas the expression of this protein was preserved in IMGN and MCD, despite severe proteinuria and foot process fusion. Also Hara *et al*. (10) reported preserved expression of SYN in a variety of glomerular diseases including MCD, IMGN, MPGN, IgA-GN and LGN. However, Srivastava *et al*. (17), while examining children with idiopathic nephrotic syndrome noticed the absence of staining for SYN in areas of sclerosis. In their study, the expression of SYN in non-sclerotic glomeruli was also significantly lower than in NK. This concerned the all groups of idiopathic nephrotic syndrome examined (MCD, diffuse mesangial hypercellularity, FSGS, and congenital nephrotic syndrome of the Finnish type). Furthermore, higher podocyte expression of SYN was associated with a significantly better response to steroid therapy (17). Similarly, Hirakawa *et al*. had shown that reduced expression of SYN in primary FSGS was associated with a worse response to steroid therapy (18). These authors suggested that deregulation of the podocyte phenotype, which is characterized by lack or reduction of podocyte-specific proteins, may contribute to poor steroid responsiveness in FSGS. In addition, they found a significant negative correlation between proteinuria and SYN expression in FSGS. We were unable to confirm the latter observation. A possible explanation for this disagreement may be a more advanced loss of podocytes in patients examined by Hirakawa *et al* (18).

We demonstrate the reduction in the expression of CR1 in proliferative and non-proliferative GN. Similar results have been published by other authors (7, 10, 15-16). However, Nolasco *et al*. (21) found an abnormal CR1 expression primarily in proliferative GN. Also Hara *et al*. (10) noticed a decreased expression of CR1 in MPGN, IgA-GN, LGN and Schoenlein-Henoch disease (SH), despite no changes in the expression of other podocyte markers (SYN, podocalyxin) observed. Supporting these data, Moll *et al*. (6) have demonstrated a decreased expression of both extracellular and intracellular tail of CR1 in IgA-GN and LGN. Colasanti *et al*. (22) have shown loss of CR1 activity in GN with capillary wall abnormalities. According to Kazatchkine and co-workers, loss of CR1 expression in glomeruli was the predominant feature of LGN class IV. The glomerular expression of CR1 was preserved in primary MesPGN, MPGN type I and II, and LGN class V. These authors suggested that loss of CR1 in LGN class IV distinguishes it both from non-proliferative LGN and other immunologically mediated proliferative GN (7).

In contrast to our results, Barisoni *et al*. (2) demonstrated a preserved expression of CR1 in non-proliferative glomerulopathies (IMGN and MCD). In their studies, the expression of CR1 in IMGN and MCD was retained at normal levels despite severe proteinuria and foot process fusion (2). However, Moll *et al*. (6), similarly to our findings, observed a decreased expression of CR1 in IMGN. In the two above cited studies, the number of patients exhibiting features of IMGN was rather small (three and five, respectively). Our group of patients with IMGN comprised 11 subjects. It may be expected that differences in CR1 expression observed in IMGN may depend on a different stage of this disease in the examined patients. This may result in a variable podocyte injury. The complement-mediated podocyte injury is suggested in IMGN (23). One of the major roles of CR1 in this disease might be to protect podocytes from this injury, since these cells do not express other complement regulatory proteins such as decay accelerating factor (DAF), monocyte chemoattractant protein (MCP), or CD59 (24). Moll and co-workers demonstrated that the drop in CR1 expression on podocytes in IMGN concerned both extra- and intracellular tails of this molecule. The authors suggested that decreased CR1 synthesis might render podocytes highly sensitive to complement attack (6). Consequently, and in line with the concept of Kazatchkine *et al*. (7) and Colasanti *et al*. (22), C3b receptors may become undetectable in many glomerular diseases with lesions of capillary walls, because of the loss of podocytes’ integrity.

We found an inverse relationship between the podocyte CR1 expression and serum creatinine level. In addition, a positive correlation between the expression of this molecule and eGFR was obtained. However, no relationship between CR1 expression and proteinuria could be stated. In this context, in the study of Hara *et al*. the expression of CR1 was related with the degree of pathological changes in the glomeruli, proteinuria and hematuria (10). Furthermore, Iida *et al*. observed that negative staining for glomerular CR1 in IgA-GN patients was associated with increased serum creatinine level (14). Altogether, these data suggest that CR1 might be a useful marker that allows not only detection of podocyte injury, but also the assessment of renal prognosis.

As in the case of SYN and CR1, we observed a dramatic decrease in the NEP expression in glomeruli with marked proliferative response of glomerular cells (ExGN) and/or advanced sclerotic lesions (FSGS). Earlier, reduction in the NEP expression was noticed in idiopathic collapsing FSGS and HIV-associated nephropathy, whereas the preserved expression of this protein was observed in IMGN and MCD (2). Sasaoka *et al*. (13) reported a decreased expression of NEP in IgA-GN, which was associated with renal dysfunction, histopathological damage and poor renal prognosis. We were unable to find any relationship between the expression of NEP and serum creatinine level, as well as between the NEP expression and protein loss in the examined patients.

Our results show that the podocyte expression of NEP is related with that of SYN and CR1. It has to be taken into account that reduced immunohistochemical staining intensity may be a spurious effect of loss of podocytes rather than reduced expression (implying less protein molecules per podocyte cell). Nevertheless, the immunohistochemical examination of podocyte markers could be very useful clinically. In this way, it would be very easy to distinguish MCD from its mimickers with a worse prognosis. The immunohistochemical examination is also much faster (and less expensive) than electron microscopy, which is required to determine the degree of podocyte loss and injury. Furthermore, the obtained negative relationship between the expression of CR1 and parameters of renal function indicates that the latter marker appears the most sensitive with respect to the prediction of renal prognosis.
